# Honeysuckle as a Food-Medicine Resource: A Review of Its Multi-Target Pharmacological Effects and Emerging Applications

**DOI:** 10.3390/molecules31162792

**Published:** 2026-08-11

**Authors:** Jie Gao, Liheng Li, Yan Li

**Affiliations:** 1 Heilongjiang University of Chinese Medicine, Harbin 150040, China; hljzyygj@outlook.com; 2 Heilongjiang Key Laboratory of Chinese Medicine Informatics, Harbin 150040, China

**Keywords:** *Lonicera japonica*, food and medicine homology, chemical composition, multi-target pharmacology, gut–brain axis, precision development

## Abstract

*Lonicera japonica* Thunb. (honeysuckle), a traditional herb with “food-medicine homology” status in Chinese medicine, is valued for its antipyretic and detoxifying properties. This review systematically summarizes its chemical composition—over 507 identified compounds, including phenylpropanoids, flavonoids, triterpenoids, saponins, and the plant-specific miR2911—as well as its multi-target pharmacological mechanisms and emerging translational applications, with particular emphasis on the gut–brain axis-mediated neuroprotective effects. Despite low oral bioavailability, honeysuckle polysaccharides and chlorogenic acid have been shown to exert significant neuroprotection in Alzheimer’s disease models by modulating gut microbiota composition, increasing short-chain fatty acid production, and restoring intestinal barrier integrity—a mechanism that challenges conventional direct-action paradigms. We also outline the applications of honeysuckle in functional foods, pharmaceuticals, animal husbandry, and cosmetics, and propose future directions including precision fermentation and mechanism-driven clinical trials. By integrating phytochemistry, pharmacology, and biotechnology, this review provides a roadmap for the evidence-based development of honeysuckle as a precise medicinal and edible resource.

## 1. Introduction

*Lonicera japonica* Flos. (honeysuckle), commonly known as honeysuckle or Jinyinhua, is a dried flower bud belonging to the Caprifoliaceae family. It has a long history of use in traditional Chinese medicine (TCM) and was documented as a superior-grade herb in the Mingyi Bielu. The Chinese Pharmacopoeia recognizes its antipyretic and detoxifying properties [1]. In China, the primary production regions for honeysuckle are Shandong, Henan, and Hebei provinces—widely recognized as its daodi (authentic) producing areas.

Recent phytochemical investigations have identified a diverse array of bioactive constituents in honeysuckle, including phenylpropanoids (e.g., chlorogenic acid, caffeic acid), flavonoids (e.g., luteoloside, rutin, quercetin), triterpenoids and saponins (e.g., hederagenin, ursolic acid, oleanolic acid), iridoid glycosides (e.g., sweroside, loganin), monoterpenes, polysaccharides, and the plant-specific microRNA miR2911 [1,2,3]. These compounds have been reported to exhibit a broad spectrum of pharmacological activities, including antibacterial, anti-inflammatory, antiviral, antioxidant, antidiabetic, cardioprotective, neuroprotective, and immunomodulatory effects [4,5,6]. Accumulating evidence suggests that the gut–brain axis may serve as a key mediator of the neuroprotective effects attributed to honeysuckle polysaccharides and chlorogenic acid. This emerging perspective offers a new framework for understanding the therapeutic potential of these compounds against neurodegenerative diseases [7,8]. Unlike conventional neuroprotective agents that act directly on central nervous system targets, this indirect mechanism—operating through microbiota modulation, short-chain fatty acid production, and intestinal barrier repair—may offer a safer and more sustainable strategy for managing chronic neurodegenerative conditions, although clinical validation remains incomplete.

In addition to its medicinal properties, honeysuckle is a nutrient-rich plant containing amino acids, vitamins, and minerals, which further supports its use as a functional food ingredient. The National Health Commission of China included honeysuckle in the “Food and Medicine Homology” catalog in April 2018, facilitating its application in the food and health product sectors [3].

To distinguish this review from previous works and to address the need for an integrated, evidence-based evaluation, four specific objectives guide its structure: (1) to provide a comprehensive overview of honeysuckle constituents—with over 507 compounds reported to date—and, where possible, to include quantitative activity data to facilitate cross-study comparisons; (2) to critically examine the gut–brain axis mechanism underlying neuroprotection; (3) to delineate evidence levels (in vitro, animal, clinical) in assessing translational potential; and (4) to bridge mechanistic findings with industrial applications across the food, pharmaceutical, veterinary, and cosmetic sectors. Through this integrated approach, this review aims to serve as a practical reference for the evidence-based development of honeysuckle as both a medicinal and an edible resource. The literature search for this review was conducted in PubMed, Web of Science, CNKI, and Google Scholar up to June 2026, and a total of 161 original research articles and 7 clinical or translational reports were included.

## 2. Chemical Composition

To date, more than 507 chemical compounds have been identified from honeysuckle, primarily consisting of phenylpropanoids, flavonoids, triterpenoids, iridoid glycosides, and monoterpenes [5].

### 2.1. Phenylpropanoids

Phenylpropanoids are among the predominant constituents of honeysuckle and include caffeoylquinic acid (CQA) derivatives. Caffeoylquinic acids (CQAs) represent a large subclass of phenolic acids; chlorogenic acid (5-O-caffeoylquinic acid) is the most abundant monomer within the CQA family, together with caffeic acid, and isochlorogenic acids A, B, and C. These compounds have been reported to possess antibacterial, anti-inflammatory, and antioxidant activities [9,10]. Table 1 summarizes the names, molecular formulas, and pharmacological properties of representative phenylpropanoids.

### 2.2. Flavonoids

Flavonoids are a class of phenolic compounds characterized by an oxygen-containing heterocyclic structure. Representative flavonoids found in honeysuckle include luteolin, rutin, quercetin, and lonicerin. Table 1 lists the names, molecular formulas, and pharmacological activities of key flavonoids.

### 2.3. Triterpenoids

Triterpenoids and their saponin derivatives identified in honeysuckle include hederagenin, macranthoidin A, macranthoidin B, oleanolic acid, and ursolic acid. These compounds have been reported to exhibit anti-inflammatory, immunomodulatory, and cardioprotective effects [1,11,12,13].

### 2.4. Iridoid Glycosides

Iridoid glycosides occur predominantly in glycosidic form in honeysuckle, with prominent examples including sweroside, loganin, and morroniside. These compounds have demonstrated a variety of pharmacological effects, including antitumor, cardioprotective, and immunomodulatory activities [2,14,15,16,17]. Detailed information on key iridoid glycosides is provided in Table 1.

### 2.5. Monoterpenes and Other Components

The volatile oils of honeysuckle are rich in various compounds and have been reported to exhibit antibacterial, antiviral, and antioxidant properties. Key monoterpene constituents include linalool, geraniol, and α-terpineol [18]. In addition, honeysuckle contains polysaccharides (LJP, LJF, etc.), phytosterols, and the plant-specific microRNA miR2911 [15,19,20,21,22]. Notably, miR2911—a microRNA found exclusively in honeysuckle—has been reported to possess both antiviral and antitumor activities [23]. Comprehensive information on the monoterpenes and other constituents is summarized in Table 1.

**Table 1:** Key Active Compounds in Honeysuckle and Their Major Pharmacological Effects.

Category	Representative Compound	Molecular Formula	Major Pharmacological Effects	References
Phenylpropanoids	Chlorogenic acid	C_16_H_18_O_9_	Anti-inflammatory, antibacterial, antiviral, antioxidant	[24,25]
Phenylpropanoids	Caffeic acid	C_9_H_8_O_4_	Antibacterial, antioxidant	[26]
Phenylpropanoids	Isochlorogenic acid A/B/C	C_25_H_24_O_12_	Anti-inflammatory, antiviral, hypoglycemic	[1]
Phenylpropanoids	Ferulic acid	C_10_H_10_O_4_	Anti-inflammatory, neuroprotective	[27]
Flavonoids	Luteoloside	C_21_H_20_O_11_	Antiviral (RSV, IAV), anti-inflammatory	[28]
Flavonoids	Rutin	C_27_H_30_O_16_	Antitumor, antioxidant, cardioprotective	[26,29]
Flavonoids	Quercetin	C_15_H_10_O_7_	Antitumor, anti-COVID-19, antidepressant	[29]
Flavonoids	Luteolin	C_15_H_10_O_6_	Antitumor, anti-inflammatory, anti-HBV	[28]
Flavonoids	Lonicerin	C_27_H_30_O_15_	Antiviral	[2]
Flavonoids	Hyperoside	C_21_H_20_O_12_	Antidepressant, neuroprotective	[30,31,32]
Triterpenoids	Hederagenin	C_30_H_48_O_4_	Immunomodulatory, anticomplement	[15,33]
Triterpenoids	Macranthoidin A	C_65_H_106_O_30_	Anti-inflammatory, immunomodulatory	[34,35,36]
Triterpenoids	Ursolic acid	C_30_H_48_O_3_	Cardiovascular protection, antitumor	[37,38]
Triterpenoids	Oleanolic acid	C_30_H_48_O_3_	Anti-inflammatory, hepatoprotective	[39,40]
Iridoid glycosides	Sweroside	C_16_H_22_O_9_	Anti-inflammatory, hepatoprotective	[2,41]
Iridoid glycosides	Morroniside	C_17_H_26_O_11_	Neuroprotective, anti-inflammatory	[2]
Iridoid glycosides	Loganin	C_17_H_26_O_10_	Anti-inflammatory, analgesic	[41]
Monoterpenes	Linalool	C_10_H_18_O	Antibacterial, anti-inflammatory, sedative	[18]
Monoterpenes	Geraniol	C_10_H_18_O	Antibacterial, antioxidant	[18]
Monoterpenes	α-Terpineol	C_10_H_18_O	Antibacterial, antiviral	[18]
Others	Honeysuckle polysaccharides (LJP/LJF)	—	Immunomodulatory, antitumor, hypoglycemic	[3,42]
Others	miR2911	—	Antiviral (HPV, HBV), antitumor	[43,44]

## 3. Pharmacological Effects and Molecular Mechanisms

**Figure 1:**
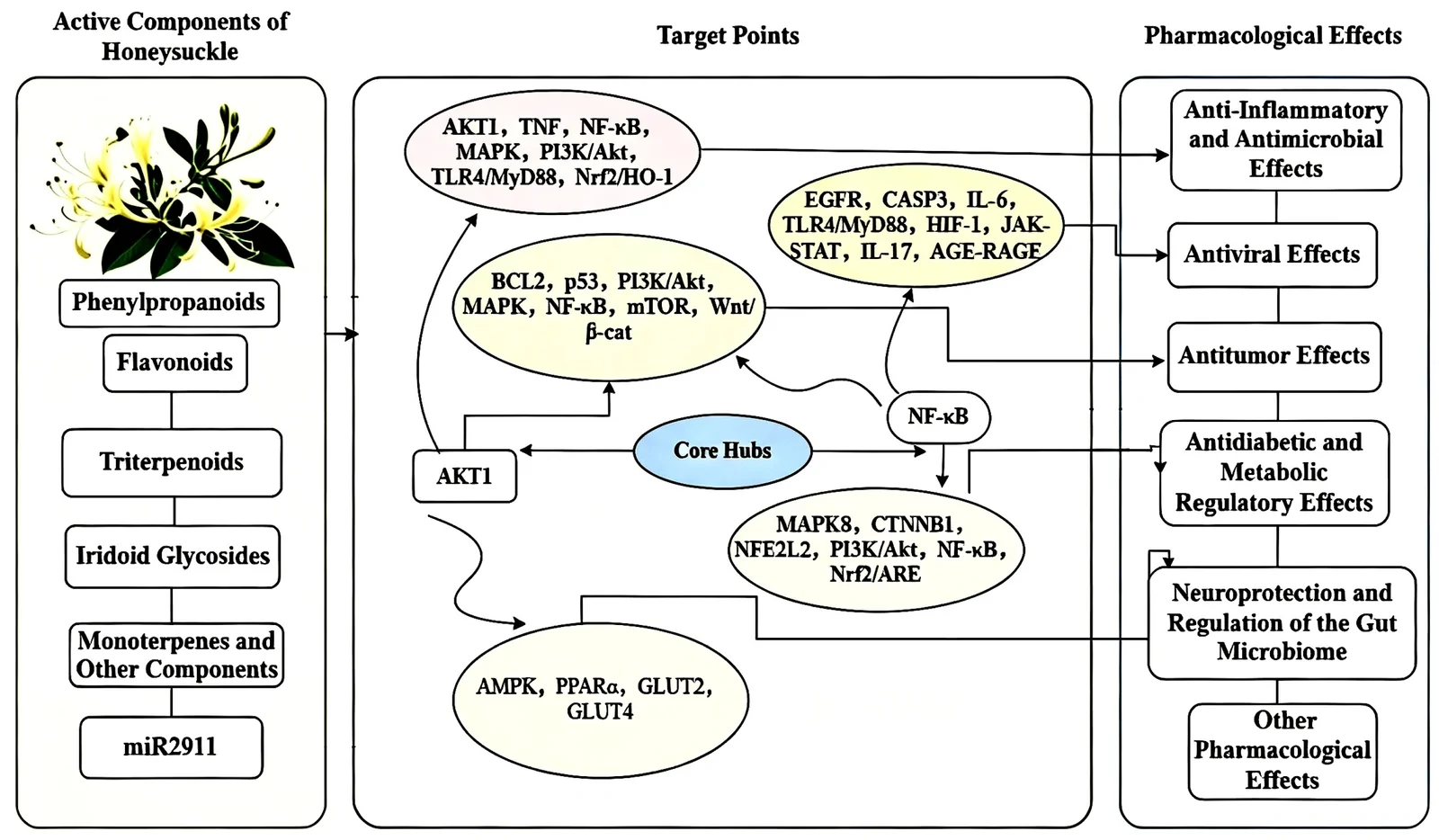
Arrows indicate activation (→), bidirectional regulation (↔). This network illustrates how active compounds modulate key targets and pathways to produce diverse pharmacological outcomes.

Honeysuckle and its constituents exert various pharmacological effects through an intricate regulatory network involving multiple components, targets, and pathways (Figure 1). Network pharmacology analyses suggest that flavonoids, phenylpropanoids, and cycloartenolides in honeysuckle collectively modulate key targets such as MAPK8, NFKB1, EGFR, and BCL2, thereby influencing signaling pathways associated with inflammation, oxidative stress, and apoptosis [6].

### 3.1. Anti-Inflammatory and Antimicrobial Effects

Honeysuckle has been reported to possess broad-spectrum antimicrobial activity against a wide range of microorganisms, including Gram-positive and Gram-negative bacteria, molds, and yeasts [3,45,46,47]. Its anti-inflammatory and antimicrobial effects are generally attributed to synergistic interactions among multiple bioactive constituents that concurrently modulate various signaling cascades, although direct experimental evidence confirming such integrated multi-component and multi-target actions remains limited. It is important to note that many of the mechanistic pathways discussed herein (e.g., NF-κB, MAPK, PI3K/Akt) have been primarily elucidated using isolated compounds in simplified in vitro systems, and their physiological relevance to the complex mixture of constituents present in honeysuckle extracts or decoctions has not been systematically validated. Network pharmacology studies have identified quercetin and luteolin as key active ingredients that target core proteins such as AKT1 and TNF, providing mechanistic clues for their anti-inflammatory and antimicrobial functions through the modulation of PI3K/Akt, MAPK, and calcium signaling pathways [48,49,50]. Importantly, while network pharmacology can generate useful hypotheses regarding multi-target interactions, these in silico predictions require experimental validation—through biochemical assays or genetic approaches—to confirm functional relevance.

Quantitative analyses have further revealed the potent anti-inflammatory activity of luteolin, a major flavonoid in honeysuckle. In an in vitro neutrophil model, luteolin inhibited fMLP-induced chemotaxis with an IC_50_ of 0.2 ± 0.1 μmol/L and suppressed the respiratory burst with an IC_50_ of 2.2 ± 0.8 μmol/L, effects attributed to the blockade of MEK/ERK and PI3K/Akt signaling pathways [51].

Chlorogenic acid, a key anti-inflammatory component, has been shown to reduce the release of pro-inflammatory mediators such as IL-8 by blocking NF-κB and MAPK pathways, scavenging reactive oxygen species (ROS), and reducing p38 MAPK phosphorylation [52,53]. Honeysuckle has also been reported to modulate the TLR4/MyD88/NF-κB pathway, suppress TNF-α and IL-6 secretion, inhibit NLRP3 inflammasome activation, and activate the Nrf2/HO-1 antioxidant pathway, collectively contributing to its anti-inflammatory effects [54,55].

Other bioactive compounds appear to exert cooperative effects. Polysaccharides have been shown to target EGFR and HSP90AA1, modulate gut microbiota balance, and contribute to immune regulation [56]. Ferulic acid acts through the regulation of Nrf2 and NF-κB pathways, while oleanolic acid and ursolic acid have been reported to reduce endothelial inflammation by inhibiting NF-κB activation [57,58,59]. Luteolin reduces the production of cytokines such as IL-1β and TNF-α [60,61]. Caffeic acid has demonstrated potent antimicrobial activity against Staphylococcus aureus, Escherichia coli, and Aspergillus flavus [62,63,64].

Clinical evidence, although limited, suggests that oral honeysuckle extract as an adjunctive therapy for acute exacerbations of chronic obstructive pulmonary disease (COPD) may improve response rates, increase serum IgA, IgM, and IgG levels, and reduce inflammatory markers such as TNF-α and CRP [65]. In addition, animal studies have shown that a honeysuckle-containing compound can reduce joint inflammation and bone erosion in rats with collagen-induced arthritis by blocking the TLR4/NF-κB signaling pathway [66], further supporting its potential therapeutic value in inflammatory disorders.

### 3.2. Antiviral Effects

Honeysuckle and its active constituents have been reported to exhibit broad-spectrum antiviral properties against various pathogens, including influenza A virus (IAV), severe acute respiratory syndrome coronavirus 2 (SARS-CoV-2), herpes simplex virus (HSV), and hepatitis B virus (HBV). Network pharmacology studies suggest that the antiviral mechanisms may involve the modulation of signaling pathways such as TLR4/MyD88/NF-κB, HIF-1, PI3K/Akt, MAPK, and Toll-like receptor pathways [54,67].

#### 3.2.1. Antiviral Activity Against Influenza

Influenza A virus (IAV) is a major global public health threat belonging to the Orthomyxoviridae family [44,68]. Several studies have reported that honeysuckle and its active components can effectively suppress IAV replication [4]. Transcriptomic analysis revealed that a 6.25 μg/mL ethanol extract of honeysuckle markedly suppressed viral nucleoprotein expression in infected macrophages and reduced pro-inflammatory cytokines including IL-6, TNF-α, and IL-1β. A total of 262 differentially expressed genes were identified, predominantly associated with pattern recognition receptor (PRR) pathways, including TLR, RLR, and NLR. Further investigations demonstrated that honeysuckle inhibits viral replication and attenuates inflammatory responses by suppressing TLR3, RIG-I, and NLRC5, thereby preventing overactivation of the PRR–NF-κB signaling pathway [67].

The anti-influenza efficacy of honeysuckle is attributed to the combined action of multiple compounds. Vogeloside and secoxyloganin, both cycloartenolides, have been identified as key anti-IAV agents [69]. miR2911, a honeysuckle-derived microRNA, has been shown to target the PB2 and NS1 genes of IAV, thereby inhibiting viral replication [44]. Caffeic acid exhibits antiviral activity by blocking neuraminidase function [70]. Animal studies have confirmed that honeysuckle extract treatment significantly reduces lung damage in IAV-infected mice [71], while chlorogenic acid has demonstrated protective effects against H9N2 avian influenza virus-induced lung injury in mouse models [44,72].

In addition to network pharmacology-predicted targets, the antiviral effects of honeysuckle have been corroborated by quantitative in vitro evidence. A mixed extract of organic acids and flavonoids from honeysuckle inhibited the replication of influenza viruses H1N1 and H3N2, with EC_50_ values of 3.8 μg/mL and 4.1 μg/mL, respectively—comparable to the antiviral activity of oseltamivir [73]. Furthermore, the ethyl acetate fraction of honeysuckle exhibited inhibitory activity against neuraminidases of H3N2 and H1N1, with IC_50_ values of 243.18 ± 18.47 μg/mL and 198.34 ± 9.67 μg/mL, respectively [74].

#### 3.2.2. Anti-COVID-19 Effects

COVID-19, caused by SARS-CoV-2, induces pathology through two primary mechanisms: direct viral damage and the cytokine storm triggered by the host immune response [75]. Network pharmacology analyses suggest that honeysuckle may exert anti-SARS-CoV-2 effects through pathways including HIF-1, PI3K/Akt, MAPK, and Toll-like receptor signaling, with EGFR and IL-2 as potential targets [76]. Honeysuckle may also have therapeutic potential for COVID-19 complications, particularly SARS-CoV-2 infection complicated by myocarditis. Its mechanism may involve luteolin and quercetin targeting AKT1, CASP3, and IL-6, as well as the AGE-RAGE and IL-17 pathways [77]. Honeysuckle-containing formulations, such as Jinye Baidu Granules, have shown promise against SARS-CoV-2, with compounds including indirubin and isorhamnetin binding to ACE2 and the SARS-CoV-2 3CL protease [78]. Additionally, quercetin and kaempferol in the Astragalus–Agastache–honeysuckle combination may exert preventive effects through the AGE-RAGE and IL-17 pathways [79].

From the perspective of active components, quercetin and β-sitosterol have been identified as key anti-COVID-19 compounds targeting molecules such as IL-6, ALB, and TNF, and may regulate viral replication, inflammatory responses, and apoptosis through AMPK, NF-κB, and JAK-STAT pathways [80,81]. Quercetin has been reported to promote regulatory T cell differentiation and downregulate alkaline phosphatase (ALP), high-sensitivity C-reactive protein (hs-CRP), and lactate dehydrogenase (LDH) levels [75]. Diosmetin modulates the M1/M2 macrophage polarization balance, while luteolin upregulates IL-10 and inhibits the TLR4/NF-κB signaling pathway, potentially alleviating acute respiratory distress syndrome-associated lung injury [71,82,83,84].

#### 3.2.3. Antiviral Activity Against Herpesviruses

Herpesviruses are enveloped double-stranded DNA viruses classified into three subfamilies (α, β, γ) based on their host range. Human herpesvirus 4, also known as Epstein–Barr virus (EBV), belongs to the γ-herpesvirus subfamily and has been strongly associated with the development of several malignancies [15,85,86,87,88].

FA15, a ferulic acid derivative, has been reported to completely inhibit EBV activation at 100 μM without cytotoxic effects at 20 μM [89]. Chlorogenic acid, a major honeysuckle component, has also demonstrated notable antiviral activity against herpes simplex virus type 1 (HSV-1) [90,91]. Both honeysuckle extract and a combined eye drop formulation containing Houttuynia cordata and honeysuckle have been shown to prevent HSV-1-induced cytopathic effects in Vero cells. Clinical investigations have reported that honeysuckle extract significantly ameliorates lesion severity and associated symptoms in patients with herpetic keratitis [92,93].

Lonicera granules have demonstrated strong in vitro inhibitory efficacy against HSV-1. Clinical trials indicate that adjunctive use of these granules alongside standard therapy for viral infections such as hand, foot, and mouth disease may reduce the duration of rash, oral herpes, and fever [94,95].

#### 3.2.4. Antiviral Activity Against Hepatitis B Virus

HBV is a circular DNA virus primarily transmitted through blood and bodily fluids and is associated with acute and chronic hepatitis, liver fibrosis, and hepatocellular carcinoma [96,97]. In vitro studies have demonstrated that total honeysuckle extract dose-dependently inhibits HBsAg and HBeAg secretion and HBV DNA replication in HepG2.2.15 cells [96,98]. A novel caffeoylquinic acid derivative, 3-caffeoylquinic acid, exhibited inhibition rates of 83.82%, 70.76%, and 39.36% for HBsAg, HBeAg, and HBV DNA, respectively, at 100 μg/mL [98,99]. Chlorogenic acid exerts a dual effect against HBV: it reduces HBV markers in HepG2.2.15 cells and decreases viral load in a duck hepatitis B virus (DHBV) model [96]. Liu et al. also reported its significant inhibitory effect on HBsAg and HBeAg secretion [100]. Isochlorogenic acid A has been shown to impair viral replication by interfering with multiple stages of the HBV life cycle and activating the HO-1-mediated antioxidant stress pathway [101].

The flavonoids luteolin and quercetin have demonstrated synergistic antiviral effects against HBV by inhibiting viral replication and reducing hepatic inflammation [28,29]. Animal studies have shown that honeysuckle extract improves liver histopathological damage in DHBV-infected ducks and reduces inflammatory infiltration [98]. Limited clinical evidence suggests that adding honeysuckle-based compound formulas to standard nucleos(t)ide analogue therapy for chronic hepatitis B may improve virological response rates and liver function [102].

### 3.3. Antitumor Effects

Honeysuckle-derived compounds, including chlorogenic acid, luteolin, quercetin, and caffeoylquinic acid derivatives, have been reported to modulate PI3K/Akt, MAPK, NF-κB, and p53 pathways, thereby inhibiting tumor cell proliferation, inducing apoptosis, and suppressing migration in preclinical models [5,103].

In hepatocellular carcinoma (HCC), caffeoylquinic acid derivatives, luteolin, quercetin, and chlorogenic acid have been shown to induce apoptosis through Bcl-2/caspase, PI3K/Akt, and p53/caspase cascades [5,104]. WLJP-03A suppresses HCCLM3 cell proliferation and migration by binding to galectin-4 and modulating the Bcl-2/Bax/caspase-3 axis [105]. Methyl 4,5-di-O-caffeoylquinate (via Keap1/Nrf2), phloroglucinol derivatives (via mTOR/EMT), and biflavonoids have also been reported to inhibit HCC cells [106,107,108].

In lung cancer models, quercetin and kaempferol have been shown to target MMP2 and TNF through PI3K-Akt, MAPK, and apoptosis pathways. Honeysuckle extracts suppress A549 cell proliferation, migration, and invasion, and induce autophagic cell death through AKT, mTOR, and MEK/ERK signaling [109]. A polyphenolic extract has been reported to inhibit NCI-H1299 cells by upregulating miR-100-5p, regulating AKT/CREB and Wnt/β-catenin, and activating caspase-3/9 and PARP [110,111,112]. Limited evidence suggests that modified Siwei Jinyinhua Decoction combined with chemotherapy may alleviate symptoms in non-small cell lung cancer (NSCLC) patients [113].

In breast cancer models, chlorogenic acid, luteolin, and quercetin have been shown to induce apoptosis through PI3K/Akt suppression and p53/caspase activation [28,29]. Honeysuckle polysaccharides regulate Bcl-2/Bax/caspase-3 directly and enhance antitumor immunity via DC/CD8^+^ T cell activation (LJP-exosome system in 4T1 mice) [114,115]. In triple-negative breast cancer (TNBC) MDA-MB-231 cells, polysaccharides and isochlorogenic acid C have been reported to inhibit proliferation and migration and induce apoptosis [116]. Chlorogenic acid upregulates Bax, p53, and caspase-3 while downregulating Bcl-2 [117,118]. Xianfang Huoming Decoction suppresses MDA-MB-231 activity through caspase-3/Bax upregulation and Bcl-xL downregulation [119].

In colorectal cancer and other malignancies, LASNB has been shown to inhibit HCT116, HCT15, and CT26 cells through receptor tyrosine kinase–MEK–ERK and NF-κB pathways [120]. Honeysuckle vine extract suppresses HCT116 and SW480 growth through p53-dependent mitochondrial apoptosis [121]. miR2911 targets TGF-β1 mRNA, enhancing T-lymphocyte infiltration [122]. Rutin induces apoptosis in cervical cancer Caski cells by targeting HPV16/18 E6/E7 [123]. Luteolin activates p53 and arrests ovarian cancer cells in G2/M [124], while total saponins have been reported to induce apoptosis in thyroid cancer TPC-1 cells through SHP2/Ras/MAPK blockade [125].

It should be emphasized that robust clinical evidence confirming the efficacy of honeysuckle compounds as primary anticancer therapies in humans is lacking. The findings summarized above are predominantly derived from preclinical models and require further validation in well-designed clinical trials.

### 3.4. Antidiabetic and Metabolic Regulatory Effects

Diabetes mellitus (DM) is a metabolic disorder characterized by chronic hyperglycemia and abnormal lipid metabolism, representing a major global health burden [126]. Honeysuckle has a long history of use in traditional medicine, and accumulating evidence suggests that its extracts and bioactive constituents exert beneficial effects on glucose and lipid homeostasis.

The hypoglycemic actions of honeysuckle are mediated through multiple mechanisms. Inhibition of intestinal α-glucosidase represents a primary pathway: honeysuckle extracts have been reported to suppress α-glucosidase activity [127], with chlorogenic acid identified as one of the key active components contributing to this effect [128]. In particular, chlorogenic acid, along with neochlorogenic acid and isochlorogenic acids A, B, and C, has been shown to delay carbohydrate digestion and reduce postprandial glucose absorption by inhibiting intestinal α-glucosidase [127,129].

Beyond digestive enzyme inhibition, honeysuckle extract has been reported to improve systemic insulin sensitivity. In obese diabetic (ob/ob) mice, it lowers fasting blood glucose and enhances insulin sensitivity by downregulating hepatic PGC-1α expression, without affecting glycemia in normoglycemic mice, suggesting selective regulatory actions [130]. Chlorogenic acid has been shown to potentiate insulin sensitivity and glucose tolerance through activation of the CTRPs–AdipoRs–AMPK/PPARα signaling axis, which upregulates the expression of key glucose-handling genes including GLUT2, GLUT4, and PCK1 [131]. In addition, honeysuckle polysaccharides have been reported to reduce blood glucose and serum insulin levels in streptozotocin (STZ)-induced diabetic rats, while concurrently increasing hepatic and muscle glycogen content and attenuating oxidative stress [132].

### 3.5. Neuroprotection and Regulation of the Gut Microbiome

#### 3.5.1. Neuroprotective Effects

Honeysuckle has emerged as a subject of interest in neuropharmacological research. Network pharmacology studies have elucidated its potential impact on pathways associated with Alzheimer’s disease (AD), demonstrating interactions with key targets including MAPK8, CTNNB1, NFKB1, EGFR, BCL2, and NFE2L2. Molecular docking analyses have confirmed the high binding affinity of compounds such as hederagenin, luteolin, and quercetin to these targets [6].

Honeysuckle has been reported to exert neuroprotective effects on neurons and glial cells at the cellular level. The extract has been shown to protect SH-SY5Y neurons from H_2_O_2_-induced apoptosis through modulation of MAPK and PI3K/Akt signaling pathways [133]. Flavonoid constituents have been reported to inhibit the PI3K/Akt/NF-κB pathway, resulting in reduced secretion of TNF-α, IL-1β, and NO from LPS-stimulated BV2 microglia, thereby alleviating neuroinflammation [134].

Chlorogenic acid has demonstrated protective effects through both direct and indirect pathways. Direct actions include activation of the Nrf2/ARE antioxidant pathway, blockade of NF-κB/p38 MAPK inflammatory signaling, and modulation of Akt/Erk1/2 and PI3K/AKT anti-apoptotic pathways. Indirect pathways involve modulation of gut microbiota and intestinal barrier function, affecting outcomes through the “gut–brain axis” [7].

Polysaccharides have been reported to activate the Nrf2 pathway, reducing oxidative stress, and to increase brain acetylcholinesterase (AChE) levels, which is associated with improved cognitive function. Additionally, polysaccharides modulate gut microbiota composition—specifically, they reduce the Firmicutes/Bacteroidetes ratio while increasing the abundance of Lactobacillaceae and Bifidobacteriaceae [8]. Flavonoids such as luteolin and quercetin have been shown to inhibit NF-κB activation in microglia, reducing the release of pro-inflammatory cytokines including IL-1β and TNF-α, and to reduce Aβ production by inhibiting BACE1 function [28,135].

In an aluminum-induced AD mouse model, honeysuckle and its compound formulation significantly enhanced cognitive function by increasing brain AChE activity, improving antioxidant capacity, and reducing pathological damage to cortical neurons [136].

#### 3.5.2. Regulatory Mechanisms of the Gut–Brain Axis

The gut–brain axis facilitates bidirectional communication through metabolic, immune, neuroendocrine, and vagal pathways [7]. Short-chain fatty acids (SCFAs), produced by gut microbiota, play a key role in linking gut health with central nervous system function [137]. SCFAs can cross the blood–brain barrier and bind to receptors on neurons and glial cells (such as GPR41 and GPR43) to modulate cellular functions [138]. They have been reported to inhibit M1 polarization of microglia, promote M2 transformation, reduce secretion of IL-1β, TNF-α, and IL-6, and enhance BDNF expression to promote synaptic plasticity, thereby exerting neuroprotective effects [137].

Honeysuckle polysaccharides and chlorogenic acid exhibit prebiotic-like properties and influence gut microbiota composition. They have been shown to enhance the abundance of beneficial bacteria such as Lactobacillus and Bifidobacterium, reduce the Firmicutes/Bacteroidetes (F/B) ratio, and increase gut microbiota diversity under conditions of stress or inflammation [138,139,140]. Moreover, honeysuckle has been reported to upregulate the expression of tight junction proteins ZO-1 and Occludin, reinforcing the intestinal mucosal barrier, reducing LPS translocation and intestinal permeability, and interrupting the pathological sequence of “leaky gut–peripheral inflammation–central neuroinflammation” [138,141].

**Figure 2:**
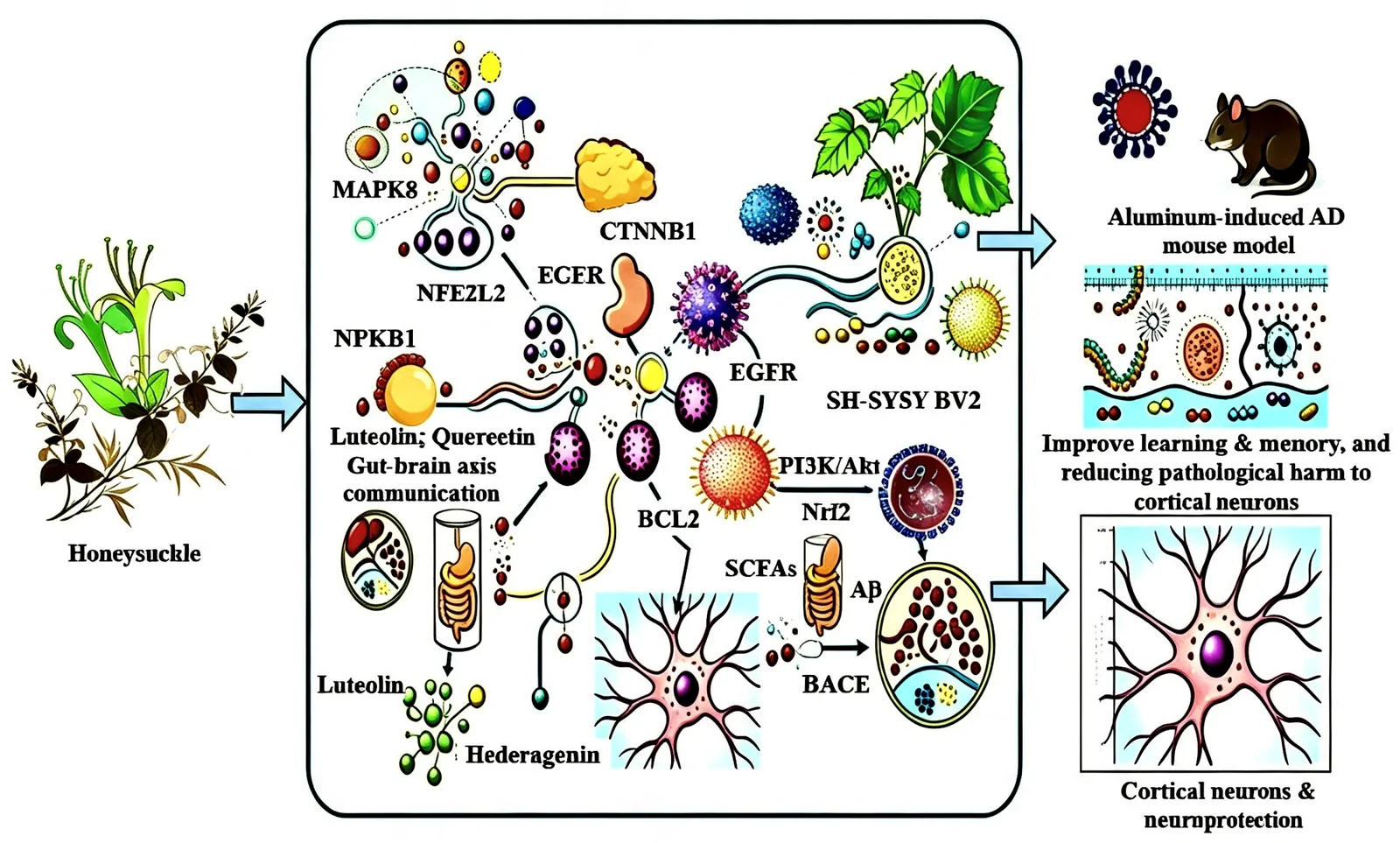
Mechanistic overview of the neuroprotective effects of *Lonicerae japonicae* Flos. Its bioactive components—luteolin, quercetin, and hederagenin—target key regulators (MAPK8, NFKB1, EGFR, BCL2, Nrf2) and signaling pathways (PI3K/Akt, Nrf2/ARE, NF-κB). This direct action helps protect neurons and microglia by inhibiting oxidative stress, neuroinflammation, and Aβ production. On the gut side, honeysuckle also modulates the gut microbiota, increases short-chain fatty acid (SCFA) levels, and reinforces the intestinal barrier. These changes support the gut–brain axis and help lower systemic inflammation. In an aluminum-induced Alzheimer’s disease mouse model, these combined effects lead to improved cognitive function, restored AChE activity, and reduced cortical neuron damage.

Honeysuckle has been shown to inhibit inflammatory pathways such as NF-κB, reducing TNF-α, IL-1β, and IL-6 levels, thereby alleviating secondary damage to brain tissue from systemic chronic inflammation [35,54]. In vitro and in vivo studies further suggest that nanovesicles derived from honeysuckle exhibit combined anti-inflammatory and neuroprotective effects by modulating gut microbiota composition, enhancing SCFA levels, and repairing intestinal barrier integrity [142]. The diverse neuroprotective and gut–brain regulatory mechanisms of honeysuckle are depicted in Figure 2.

### 3.6. Other Pharmacological Effects

In addition to the aforementioned activities, honeysuckle has been reported to exhibit a wide range of pharmacological effects, including immunomodulatory, cardioprotective, anti-aging, and antidepressant properties. Hederagenin and its derivatives have been shown to exhibit anti-complement activity [15]. Ursolic acid has been reported to exert cardioprotective effects in rat heart tissue by reducing H_2_O_2_ production in isolated cardiac mitochondria and uncoupling mitochondrial oxidative phosphorylation, effects resembling myocardial ischemic preconditioning [143]. Furthermore, ursolic acid has been reported to improve vascular endothelial function [144].

Chlorogenic acid, which is abundant in honeysuckle, contains an R–OH group in its molecular structure that can effectively scavenge reactive oxygen species such as superoxide anions (O_2_^−^·) and hydroxyl radicals (·OH). This antioxidant activity may help reduce free radical-induced cellular damage, protect erythrocyte membranes, and mitigate ultraviolet-induced oxidative stress, thereby contributing to anti-aging effects [24]. An ethanolic extract of honeysuckle significantly reduced the number of writhing episodes and prolonged writhing latency in a mouse acetic acid writhing model, indicating analgesic activity [145]. Quercetin has been reported to exert antidepressant effects by activating the tyrosine kinase receptor B (TrkB)/β-catenin signaling pathway [29]. The honeysuckle-derived polysaccharide LJP-1 has been shown to lower serum uric acid through xanthine oxidase inhibition and attenuate sodium urate crystal-induced ankle joint edema, suggesting potential therapeutic value for hyperuricemia [146].

In terms of cardiovascular protection, honeysuckle and its phenylpropanoid constituents have demonstrated antiplatelet aggregation activity. Among these, caffeic acid and isochlorogenic acid derivatives (such as 3,4-dicaffeoylquinic acid) exhibited particularly prominent effects, with IC_50_ values as low as 0.026 g·L^−1^ [147].

## 4. Modern Applications

Honeysuckle, classified in TCM as a “food and medicine of the same origin” (shiyao tongyuan) herb, has evolved from its traditional use in heat-clearing and detoxification to various modern applications in functional foods, pharmaceuticals, and animal health. The products listed in Table 2 and Table 3 were selected based on the following criteria: (1) registration with the National Health Commission of China (or equivalent regulatory body) as either a health food or pharmaceutical preparation; (2) honeysuckle listed as a primary or significant ingredient; and (3) availability of publicly accessible product information. This selection is intended to be illustrative rather than exhaustive, reflecting the commercial landscape in China rather than a systematic market survey.

### 4.1. Development of Functional Foods

Honeysuckle is recognized as a dual-purpose plant with both medicinal and edible applications, and its safety profile is generally considered favorable. It is commonly processed into consumer products such as teas, beverages, chewable tablets, and candies—using either the raw herb or its extract—for purposes related to inflammation, oxidative stress, and immune function. In recent years, an increasing number of health food products containing honeysuckle have become available on the Chinese market. An overview of representative product types is provided in Table 2.

It should be noted that the health benefits claimed for the products in Table 2 are primarily based on the traditional uses of their constituent herbs or compositional standards rather than rigorous clinical validation. While some products have supporting evidence from animal studies (as indicated in the “Health Benefits” column), to the best of our knowledge, none of these products have been evaluated in published randomized controlled trials to confirm their efficacy in human populations. Their regulatory approval as “health foods” in China does not require the same level of clinical evidence as pharmaceutical drugs, and readers should interpret these health claims with appropriate caution.

**Table 2:** Health care products related to honeysuckle.

Product Name	Health Benefits	Active Ingredients/Characteristic Component Content	Approval Number
Hongrun Brand Honeysuckle and Sterculia Seed Granules	Clears the throat and moistens the vocal cords (throat relief)	Per 100 g: chlorogenic acid 21.8 mg, total flavonoids (as rutin) 52 mg	National Health Food Approval No. G20030038
Yun Yisheng^®^ Honeysuckle, Prepared Rhubarb, and Adzuki Bean Capsules	Helps improve acne	Per 100 g: total anthraquinones 0.5 g, chlorogenic acid 0.73 g	National Health Food Registration No. G20240064
Fusheng Brand American Ginseng, Jujube, and Honeysuckle Drink	Helps boost immunity (based on animal studies)	Per 100 mL: total saponins 15 mg, chlorogenic acid 1.8 mg	National Health Food Registration No. G20200500
Shengqiao Brand Honeysuckle, Reishi Mushroom, and Green Tea Capsules	Antioxidant	Per 100 g: crude polysaccharides 3.53 g, polyphenols 24.2 g, chlorogenic acid 10.7 g	National Health Food Registration No. G20060133
Yirentian^®^ Papaya and Honeysuckle Tablets	Provides auxiliary protection against chemically induced liver damage (based on animal studies)	Per 100 g: oleanolic acid 101 mg, total flavonoids 284 mg	National Health Food Registration No. G20050854
Shunxin Brand Eclipta Prostrata, Honeysuckle, and Reishi Mushroom Capsules	Provides auxiliary protection against chemically induced liver damage (based on animal studies)	Per 100 g: total flavonoids 2.5 g, chlorogenic acid 6.0 g	National Health Food Registration No. G20200082
Hehui Brand Honeysuckle, Chrysanthemum, and Green Tea Extract Powder	Boosts immunity	Per 100 g: chlorogenic acid 0.5 g, tea polyphenols 4.0 g	National Health Food Registration No. G20140160
Baobei Brand Honeysuckle and Salvia Miltiorrhiza Pearl Capsules	Helps improve acne	Per 100 g: total flavonoids 100 mg	National Health Food Registration No. G20240220
Hehui Brand Honeysuckle and Chrysanthemum Herbal Tea Granules (Orange Flavor)	Boosts immunity	Per 100 g: chlorogenic acid 0.4 g, tea polyphenols 4.0 g	National Health Food Registration No. G20110710
Qingyuan Gong Brand Sichuan Fritillary Bulb, Honeysuckle, and Sterculia Fruit Drink	Soothes the throat; boosts immunity (based on animal studies)	Per 100 mL: chlorogenic acid 3.0 mg	National Health Food Registration No. G20130232
Tianqi Brand Honeysuckle and North Ginseng Lozenges	Soothes the throat	Per 100 g: chlorogenic acid 450 mg	National Health Food Registration No. G20130048
Hancaotang Brand Honeysuckle, Ophiopogon, and Schisandra Lozenges	Soothes the throat	Ophiopogon extract, honeysuckle extract, Schisandra extract, licorice extract, natural menthol	National Health Food Registration No. G20140604
Junhui Brand Honeysuckle and Luo Han Guo Drink	Throat clearing	Honeysuckle, Luo Han Guo, citric acid, stevioside, honeysuckle flavoring, purified water	National Health Food Registration No. G20190512
Zhuyuan Jikang Brand Poria, Honeysuckle, and Jujube Granules	Enhances immunity (based on animal studies)	Per 100 g: crude polysaccharides 1.7 g, total flavonoids 0.3 g	National Health Food Registration No. G20200448
Bailanglin R Propolis and Honeysuckle Extract Candy	Throat soothing	Propolis, honeysuckle extract, menthol, citric acid, isomalt, maltitol solution (75%), polyethylene glycol 6000, lemon flavoring	National Health Food Registration No. G20140810
Century Sunshine Xing’er Lozenges	Anti-fatigue; throat clearing and soothing	Per 100 g: total saponins (as ginsenoside Re) 1.71 g, total polysaccharides (as glucan) 15.0 mg	Health Food Registration No. (2002) 0430

### 4.2. Applications in Pharmaceutical Formulations

According to TCM theory, honeysuckle is characterized by a sweet flavor and cold property and is recognized for its efficacy in reducing fever and detoxifying the upper jiao. It is widely used in clinical settings as a heat-clearing and detoxifying herb. According to the Compendium of Materia Medica, it possesses the capacity to disperse heat and eliminate toxins [1]. Network pharmacology studies have revealed that honeysuckle, when combined with forsythia (Forsythia suspensa), may exert synergistic anti-influenza effects through the modulation of multiple components, pathways, and targets [148]. Yinqiao San, a classic formula containing honeysuckle, forsythia, mint, and other ingredients, is a prominent formulation for detoxification and heat-clearing, commonly prescribed for wind-heat common cold, sore throat, and upper respiratory tract infections. Honeysuckle in this formulation has been reported to effectively relieve symptoms such as fever and sore throat with a favorable safety profile. Detailed formulation data are provided in Table 3.

The pharmaceutical preparations listed in Table 3 represent a diverse array of TCM formulas that incorporate honeysuckle as a key ingredient. However, the clinical evidence supporting these products varies considerably. While some formulations—such as honeysuckle oral solution for acute tonsillitis—have been evaluated in clinical studies [66], the majority of these products appear to be approved based on historical use and compositional quality standards rather than modern randomized controlled trial data. For conditions including psoriasis, hepatitis, and cancer (e.g., Jinpu Capsules), the evidence remains largely anecdotal or derived from uncontrolled observational studies. This disparity between widespread clinical use and limited high-quality evidence represents a significant gap that merits attention in future research.

### 4.3. Applications in Animal Husbandry

Honeysuckle has shown promise as a natural alternative to feed antibiotics due to its natural origin, multi-target action, and low residue profile. In livestock and poultry, dietary supplementation with honeysuckle has been reported to significantly increase serum levels of IgG, IgA, and IgM, enhance vaccine efficacy, and reduce disease incidence [149].

Oxidative stress significantly affects livestock and poultry health and production. Honeysuckle components, including chlorogenic acid, flavonoids, and polysaccharides, exhibit potent antioxidant properties [150,151]. Studies have demonstrated that supplementing feed with honeysuckle extract increases superoxide dismutase (SOD), glutathione peroxidase (GSH-Px), and catalase (CAT) activity in animal serum and liver, while reducing malondialdehyde (MDA) levels, thereby mitigating oxidative stress damage and improving stress resistance in animals [149,152]. Additionally, honeysuckle extract has been reported to exert preventive and therapeutic effects against viral infections such as Newcastle disease virus, reducing tissue damage and mortality post-infection [153]. Incorporation of honeysuckle branch and leaf powder into swine diets has been shown to enhance finishing pigs’ daily weight gain, reduce the feed-to-weight ratio, and improve economic productivity [154].

### 4.4. Applications in Cosmetics and Personal Care Products

Honeysuckle extract is widely used in cosmetics and personal care products, owing to its antioxidant, anti-inflammatory, and antibacterial properties. Common product categories include facial cleansers, toners, face masks, and toothpastes, serving purposes such as acne treatment, anti-inflammation, skin soothing, antioxidant protection, skin brightening, and oral hygiene.

A nanoformulation of honeysuckle–dandelion extract has been reported to exhibit considerable activity against Cutibacterium acnes [155]. Furthermore, a chrysoeriol derivative from honeysuckle has been shown to reduce pro-inflammatory cytokines such as IL-6, IL-1β, and TNF-α, suggesting potential applications in wound healing, skin brightening, UV protection, and anti-aging [156]. Chlorogenic acid, another honeysuckle component, effectively absorbs UV light in the 280–350 nm range; for instance, a sunscreen formulation containing 2.15% chlorogenic acid was reported to achieve an SPF of 15 in in vitro tests [157].

A nanoemulsion delivery system using polyglyceryl fatty acid esters has been developed to improve the skin penetration of active compounds. This system has an encapsulation efficiency of 74.32% and a particle size of approximately 89.8 nm, enabling effective incorporation of phenolic and flavonoid compounds from honeysuckle [158].

## 5. Discussion

With its pharmacological characteristics of “multi-component synergy, multi-target integration, and multi-pathway crosstalk,” honeysuckle holds broad prospects for application in modern medicine and the health industry. Nevertheless, addressing the limitations of traditional applications and facilitating a transition from empirical use to precision-oriented development remains a central challenge. In light of recent research trends, the following directions warrant further exploration.

### 5.1. From Component Analysis to Mechanism Elucidation: The Emergence of Gut–Brain Axis Research

Traditional studies on honeysuckle have primarily focused on in vitro activity screening; however, the apparent contradiction between its low in vivo bioavailability and significant pharmacological effects has long been a subject of debate. The “microbiota–gut–brain axis” theory offers a new perspective for resolving this apparent paradox [7]. Studies have shown that although honeysuckle polysaccharides and chlorogenic acid cannot directly cross the blood–brain barrier, they significantly improve cognitive function in Alzheimer’s disease model mice by modulating gut microbiota composition, increasing SCFA production, and restoring intestinal barrier integrity. This suggests that the neuroprotective effects of honeysuckle may rely more on indirect regulation of the gut microbiome than on the traditionally assumed direct antioxidant or anti-inflammatory actions.

Research on other natural medicines may provide methodological reference for these investigations. Gingerol-rich ginger extracts have been shown to simultaneously regulate intestinal flora composition, metabolic profiles, and neuropathological gene expression [159]. Similarly, flavonoids extracted from winter jasmine have been reported to alleviate cognitive impairment in aging mice by increasing the abundance of beneficial bacteria and accelerating SCFA synthesis [160]. These findings provide both a technical foundation and a practical research direction for exploring honeysuckle’s effects through the microbiota–metabolite–brain axis. Future studies could combine 16S rRNA sequencing, metabolomics, and targeted neural pathway analysis to systematically investigate the underlying mechanisms.

### 5.2. From Traditional Extraction to Bioconversion: Breakthroughs in Fermentation Technology

Traditional decoction methods face limitations such as low extraction yields and poor absorption of macromolecular components. Microbial fermentation technology has been shown to significantly enhance the bioactivity and utilization efficiency of honeysuckle. Co-fermentation of honeysuckle and cassia seed extracts with Bacillus subtilis and Lactobacillus acidophilus increased total polysaccharides, flavonoids, and saponins by 275%, 72%, and 62%, respectively, compared to traditional decoction. Furthermore, in an alcohol-induced liver injury model, the hepatoprotective effect of the fermented extract was comparable to that of silymarin [161]. The core advantages of this approach are that B. subtilis degrades plant cell walls, while L. acidophilus converts macromolecules into easily absorbable small molecules, while simultaneously improving the bitter and astringent taste.

**Table 3:** Pharmaceutical preparations containing honeysuckle.

Product Name	Implementation Standard	Efficacy	Indications	Formulation
Yinju Qingyan Granules	WS-11119(ZD-1119)-2002-2012Z [162]	Promotes fluid production and quenches thirst; cools and reduces fever	Summer heat-induced restlessness and thirst, sore throat caused by ascending deficient fire	Honeysuckle, chrysanthemum, and 3 other ingredients
Yinlong Qinggan Tablets	WS-10267-(ZD-0267)-2002 [163]	Clears heat and drains dampness; regulates the liver and promotes bile secretion	Acute jaundice-associated hepatitis caused by liver and gallbladder damp-heat	Honeysuckle, *Artemisia capillaris*, gentiana, *Centella asiatica*
Ge-Bong Compound	WS3-B1035-91 [164]	Pungent-cool exterior-releasing formula	Wind-heat common cold, headache, fever, cough, sore throat	Pueraria root, *Arctium lappa* seeds, honeysuckle, and 1 other ingredient
Psoriasis Relief Granules	YBZ10512005-2009Z [165]	Dispels wind and dries dampness; clears heat and detoxifies; promotes blood circulation and removes blood stasis	Psoriasis	*Sophora flavescens*, licorice, honeysuckle, and 6 other ingredients
Appendicitis Relief Tablets	WS3-B-0440-90 [166]	Clears heat and detoxifies; disperses blood stasis and reduces swelling	Acute and chronic appendicitis	Honeysuckle, isatis leaf, *Patrinia*, dandelion, and 5 other ingredients
Yantongning Spray	WS-5642(B-0642)-2002 [167]	Dispels wind, clears heat, detoxifies, and soothes the throat	Relieves sore throat and dry throat	Amla juice, honeysuckle, platycodon, wild chrysanthemum, menthol
Jingui Wash	WS-5148(B-0148)-2012Z [168]	Clears heat, removes dampness, and relieves itching	Relieves vulvar itching and excessive vaginal discharge	*Cnidium monnieri*, *Sophora flavescens*, *Angelica sinensis*, honeysuckle, and 4 other ingredients
Compound Luo Han Guo Lozenges	WS-5129(B-0129)-2002 [169]	Nourishes yin, moistens the lungs, soothes the throat, and relieves pain	Sore throat, dry throat, dry cough, scanty sputum	Luo Han Guo, honeysuckle, *Terminalia chebula*, *Scrophularia ningpoensis*, *Asarum sieboldii*, peppermint oil
Compound Daqingye Injection	WS3-B-3901-98 [170]	Clears epidemic heat and detoxifies	Japanese encephalitis, acute and chronic hepatitis, influenza, mumps	Daqingye, honeysuckle, *Notopterygium*, *Scrophularia*, rhubarb
Huashe Jieyang Tablets	YBZ09392008 [171]	Dispels wind and clears heat; cools the blood and relieves itching	Pruritus due to blood heat and excessive wind	Qidaogu, *Zaocys dhumnades*, *Phellodendron*, honeysuckle, forsythia, and 11 other ingredients
Kubai Anti-Itch Wash	WS-6016(B-1016)-2002 [172]	Clears heat and detoxifies; removes dampness and relieves itching	Vaginal itching and excessive vaginal discharge caused by damp-heat descending	*Sophora flavescens*, *Phellodendron*, *Smilax glabra*, *Cnidium monnieri*, *Artemisia capillaris*, honeysuckle
Shuanghua Caoshanhuo Lozenges	WS-5402(B-0402)-2012Z [173]	Dispels wind and clears heat; soothes the throat and relieves pain	Relieves dryness, burning sensation, pain, and hoarseness in the throat	Honeysuckle, mint, menthol
Pediatric Antipyretic Suppositories	WS3-B-1281-93 [174]	Antipyretic, anti-inflammatory	Fever in children associated with common cold and upper respiratory tract infections	*Scutellaria baicalensis* extract, *L. japonica* extract, anakin
Zhixie Li Granules	WS3-B-2095-96 [175]	Astringent, antidiarrheal, detoxifying, and digestive	Damp-heat diarrhea, food-induced diarrhea	Myrica root, saposhnikovia root, hawthorn, honeysuckle
Shule Ointment	WS-5012(B-0012)-2002 [176]	Clears heat and dampness; relieves wind and itching	Adjuvant treatment for alleviating skin itching	*Sophora flavescens*, honeysuckle, astragalus, *Sanguisorba officinalis*, white alum
Compound Daqingye Granules	WS3-B-2747-97 [177]	Clears heat and detoxifies; relieves exterior symptoms and dispels wind	Wind-heat common cold and influenza	Daqingye, honeysuckle, rhubarb, *Scrophularia*, *Notopterygium*
Compound Shuangjin Hemorrhoid Ointment	WS-5608(B-0608)-2002 [178]	Clears heat and detoxifies; reduces swelling and relieves pain	Relieves swelling and pain caused by hemorrhoids	Prickly ash, honeysuckle, rhubarb, gardenia, golden dogspine
Pediatric Cough and Asthma Relief Effervescent Granules	YBZ05622008 [179]	Promotes lung function, relieves cough, and alleviates wheezing	Cough caused by upper respiratory tract infections	Ephedra, honeysuckle, bitter apricot kernel, isatis root, gypsum, licorice, *Trichosanthes*
Cold and Cough Granules	WS3-B-0652-91 [180]	Clears heat and detoxifies; relieves cough and resolves phlegm	Common cold with fever, headache, and cough	Honeysuckle, loquat leaf, stemona, platycodon, *Trichosanthes*, eucalyptus oil
Nasyuan Capsules	WS-10075(ZD-0075)-2002-2012Z [181]	Clears heat and toxins; unblocks nasal passages	Chronic rhinitis and sinusitis	Xanthium seeds, magnolia flower, honeysuckle, wild chrysanthemum, rubia
Yinhuang Lozenges	YBZ13582009 [182]	Clears heat and detoxifies; reduces inflammation	Acute and chronic tonsillitis, pharyngitis, upper respiratory tract infections	Honeysuckle extract, *Scutellaria baicalensis* extract
Koujie Mouthwash	WS-11345(ZD-1345)-2002 [183]	Clears heat and detoxifies	Redness, swelling, and pain in the mouth and throat	Honeysuckle, chrysanthemum, isatis root, menthol, eucalyptus oil
Honeysuckle Soft Capsules	YBZ08122009 [184]	Clears heat and detoxifies	Fever, thirst, sore throat, heat-related boils and sores	Honeysuckle, *Lonicera japonica* vine
Compound Redroot Grass Tablets	WS3-B-0340-90 [185]	Clears heat and detoxifies	Acute pharyngitis, tonsillitis, enteritis, dysentery	Redroot grass, *Houttuynia cordata*, honeysuckle, wild chrysanthemum, *Andrographis paniculata*
Bone Spur Pain Relief Liquid	WS3-B-3466-98 [186]	Dispels wind, unblocks meridians, promotes blood circulation, and relieves pain	Aching, swelling, numbness, and pain caused by osteophytes in the cervical/lumbar spine and limb joints	Black plum, *Chuanxiong*, aconite (water-processed with honeysuckle and licorice), safflower, and 7 other ingredients
Mailuoning Injection	WS3-B-3462-98-2011 [187]	Clears heat and nourishes yin; promotes blood circulation and removes blood stasis	Thrombotic occlusive vasculitis, atherosclerotic occlusive disease, cerebral thrombosis and sequelae	*Achyranthes*, *Scrophularia*, *Dendrobium*, honeysuckle
Jinpu Capsules	Pharmacopoeia of the People’s Republic of China (2015, Part I) [188]	Clears heat and detoxifies; reduces swelling and relieves pain; tonifies qi and resolves phlegm	Advanced gastric or esophageal cancer with phlegm-dampness obstruction and qi stagnation with blood stasis	Honeysuckle, centipede, processed pangolin scale, toad venom, processed myrrh, dandelion, processed frankincense, and 12 other ingredients
Bovine Bile Toxin-Resolving Tablets	Pharmacopoeia of the People’s Republic of China (2015, Part I) [188]	Detoxifies and reduces swelling; disperses nodules and relieves pain	Ulcers, mastitis, redness, swelling, and pain	Bovine bile, honeysuckle, and 5 other ingredients

This strategy has multiple industrial implications: (1) increasing the in vivo exposure of components with low bioavailability; (2) potentially generating new active metabolites, broadening the spectrum of benefits; and (3) improving palatability, laying the foundation for functional food development. Future research should elucidate the dynamic interactions among “strain–substrate–metabolite” and explore precision fermentation processes for the targeted enrichment of specific components.

### 5.3. From Bud Dependence to Comprehensive Resource Utilization: Development of Non-Traditional Parts and Secondary Components

Currently, the development and utilization of honeysuckle are heavily concentrated on flower buds, while research on “minor” components such as stems, leaves, polysaccharides, and iridoid glycosides remains limited. Notably, chlorogenic acid concentrations in honeysuckle leaves are comparable to those in the flower buds, and the leaf resource is considerably more abundant. Triterpenoid saponins in the stems possess unique advantages in anti-inflammatory and immunomodulatory effects.

The industrial development of honeysuckle in Tongwei, Gansu Province, offers valuable lessons for the broader industry. A standardized honeysuckle cultivation base covering 150,000 mu (approximately 10,000 hectares) has been established, and 32 daily chemical products have been developed on this basis. Testing results showed that chlorogenic acid levels in Tongwei honeysuckle reached 4.5%—three times higher than the Chinese Pharmacopoeia standard—while luteoloside content was 0.08%, 1.6 times the national standard. This integrated full-industry-chain approach, encompassing standardized cultivation, intensive processing, and branded operations, has effectively achieved high-value utilization of honeysuckle resources and set a practical example for the industry.

### 5.4. From Single Function to Targeted Applications: Clinical Translation and Product Differentiation

Although honeysuckle exhibits a wide range of pharmacological activities, its broad-spectrum action often results in relatively diffuse therapeutic targeting. Current clinical investigations remain largely confined to conventional anti-inflammatory and antiviral applications. Notably, high-quality randomized controlled trials targeting metabolic disorders (such as diabetes and hyperlipidemia) and neurodegenerative diseases (including Alzheimer’s disease) are severely lacking.

A clinical study evaluating honeysuckle oral solution combined with antibiotics for acute suppurative tonsillitis found that this combination significantly reduced inflammatory markers and increased serum immunoglobulin levels. These outcomes provide supporting evidence for the use of honeysuckle as an adjunctive therapeutic agent in clinical practice.

Future product development should avoid low-level homogeneous competition and instead adopt more precise and application-oriented positioning strategies. In the antiviral field, researchers could focus on developing nasal sprays and oral dissolving films for post-exposure prevention against influenza and COVID-19. For metabolic health, functional foods targeting prediabetes could be designed to regulate postprandial blood glucose primarily by inhibiting α-glucosidase activity. In gut health, synbiotic products could be developed by leveraging the prebiotic functions of honeysuckle polysaccharides. In personal care, the anti-inflammatory properties of chrysoeriol make it suitable for soothing and repairing products for sensitive skin. In oral care, the broad-spectrum antimicrobial activity of honeysuckle extracts could be used to develop biological toothpastes for periodontal pathogen control.

### 5.5. From Empirical Cultivation to Smart Agriculture: Scientific Elucidation of the Environment–Quality Relationship

The quality of honeysuckle can be influenced by multiple factors, including light, temperature, soil conditions, and harvest timing. However, the quantitative relationships between environmental factors, secondary metabolite synthesis, and pharmacological activity remain largely unknown. Honeysuckle grown in the Tongwei area has been found to contain substantially higher levels of chlorogenic acid and luteolin than the current pharmacopoeia standards require. This phenomenon suggests that certain environmental stress conditions—including drought stress and large diurnal temperature variations—may activate the phenylpropanoid metabolic pathway, thereby promoting the accumulation of phenolic acids in honeysuckle.

Future research should integrate IoT-based environmental monitoring, metabolomics, and machine learning to construct an “environment–composition–activity” predictive model, enabling digital delineation of authentic production areas and precise regulation of cultivation conditions.

### 5.6. Research Limitations and Outlook

Several methodological and evidence-related limitations should be acknowledged. High-quality pharmacokinetic studies remain scarce, and rigorous clinical trials are largely lacking, with most evidence confined to cellular and animal models.

Beyond these general gaps, specific inconsistencies in the literature merit attention. Although chlorogenic acid is widely used as a quality marker, its content does not consistently correlate with bioactivity, suggesting that other constituents or synergistic effects may be equally important. Additionally, network pharmacology predictions of key targets such as NF-κB and MAPK often require concentrations that exceed achievable plasma levels in vivo, raising questions about their clinical relevance after oral administration.

From an application perspective, most products listed in Table 2 and Table 3 are only available in China, limiting their direct relevance to Western clinical practice, while industrial development remains largely homogenized with limited high-value differentiation.

Future research should prioritize: (1) integrating gut microbiota analysis, metabolomics, and organoid models to clarify indirect mechanisms; (2) conducting rigorous randomized controlled trials for specific indications such as viral respiratory infections and pre-diabetes; and (3) advancing fermentation and nano-delivery technologies alongside full-industry-chain models to translate resource advantages into competitive health-industry products. Among the proposed applications, those with reasonable preclinical support include functional foods for blood glucose management (α-glucosidase inhibition, animal models) and antiviral formulations (in vitro and limited animal data). In contrast, applications in neurodegenerative diseases, despite mechanistic plausibility, remain speculative due to the lack of human data; precision fermentation and AI-driven cultivation models are also future perspectives rather than evidence-supported applications.

### 5.7. Barriers to Clinical Translation

Despite extensive preclinical data, translating honeysuckle into clinical applications faces major obstacles related to its material properties and evidence quality. Many active compounds, including chlorogenic acid and luteolin, suffer from poor oral bioavailability due to gut microbiota metabolism and first-pass hepatic effects, raising unresolved questions about whether parent compounds or their metabolites mediate in vivo activity. Additionally, no consensus exists on appropriate quality control markers; chlorogenic acid, the current standard, does not consistently correlate with overall bioactivity. Phytochemical variability across geographic origins, harvest times, and processing methods further complicates reproducibility and regulatory acceptance. Safety and toxicity data for honeysuckle extracts are surprisingly limited, particularly for long-term use. Although acute toxicity studies in rodents have reported a favorable safety profile, chronic toxicity, genotoxicity, and potential herb–drug interactions remain inadequately characterized [5].

The evidence base itself also presents serious limitations. Much of the published literature lacks positive controls, blinded designs, adequate sample sizes, or effect size reporting, undermining reliability and preventing meta-analysis. Regulatory ambiguity arising from honeysuckle’s dual classification as food and medicine further hinders international standardization. Addressing these barriers will require harmonized analytical protocols, physiologically relevant pharmacokinetic studies, and rigorous trials designed to handle the complexity of herbal extracts. Among the numerous compounds identified, a distinction should be made between abundance-based markers and activity-based markers. Chlorogenic acid, although widely used as a quality control standard due to its high content and stability, exhibits only moderate pharmacological potency. In contrast, luteolin and quercetin show considerably higher activity (IC_50_ values in the low micromolar range) but are present at much lower concentrations, raising questions about their contribution to the overall bioactivity of crude extracts. This discrepancy underscores the need for a more rational approach to marker selection that reflects both abundance and potency.

## Data Availability

No new data were created or analyzed in this study. Data sharing is not applicable to this article.

## Abbreviations

3C-like protease, 3CLpro; Acetylcholinesterase, AChE; Alzheimer’s disease, AD; AMP-activated protein kinase, AMPK; B-cell lymphoma 2, Bcl-2; BCL2-associated X protein, Bax; Brain-derived neurotrophic factor, BDNF; Caffeoylquinic acid, CQA; Catalase, CAT; Chronic obstructive pulmonary disease, COPD; Coronavirus disease 2019, COVID-19; C-reactive protein, CRP; Cyclic adenosine monophosphate response element-binding protein, CREB; Dendritic cell, DC; Duck hepatitis B virus, DHBV; Epithelial–mesenchymal transition, EMT; Epstein–Barr virus, EBV; Estrogen receptor, ER; G-protein-coupled receptor 41, GPR41; G-protein-coupled receptor 43, GPR43; Glutathione peroxidase, GSH-Px; Heat shock protein 90 alpha family class A member 1, HSP90AA1; Hepatitis B virus, HBV; Hepatocellular carcinoma, HCC; Herpes simplex virus type 1, HSV-1; High-performance liquid chromatography, HPLC; Human epidermal growth factor receptor 2, HER2; Immunoglobulin A, IgA; Immunoglobulin G, IgG; Immunoglobulin M, IgM; Influenza A virus, IAV; Infrared spectroscopy, IR; Interleukin-1β, IL-1β; Interleukin-6, IL-6; Interleukin-8, IL-8; Interleukin-10, IL-10; Kelch-like ECH-associated protein 1, Keap1; Lactate dehydrogenase, LDH; Lipopolysaccharide, LPS; *Lonicera japonica* polysaccharide, LJP; Malondialdehyde, MDA; Mitogen-activated protein kinase, MAPK; Mitogen-activated protein kinase 8, MAPK8; Myeloid differentiation primary response 88, MyD88; NACHT, LRR and PYD domains-containing protein 3, NLRP3; National Health Commission of China, NHC; Neuraminidase, NA; NF-κB inhibitor, IκB; Nitric oxide, NO; NLR family CARD domain containing 5, NLRC5; Non-small cell lung cancer, NSCLC; Nuclear factor erythroid 2-related factor 2, Nrf2; Nuclear factor kappa-light-chain-enhancer of activated B cells, NF-κB; Pattern recognition receptor, PRR; Phosphatidylinositol 3-kinase, PI3K; Progesterone receptor, PR; Protein kinase B, AKT (also known as PKB); Reactive oxygen species, ROS; Respiratory syncytial virus, RSV; Retinoic acid-inducible gene I, RIG-I; Runt-related transcription factor, RUNX; Severe acute respiratory syndrome coronavirus 2, SARS-CoV-2; Short-chain fatty acid, SCFA; Signal transducer and activator of transcription, STAT; Small hairpin RNA, shRNA; Sodium dodecyl sulfate–polyacrylamide gel electrophoresis, SDS-PAGE; Superoxide dismutase, SOD; T helper cell, Th; Tight junction protein, ZO-1 (zonula occludens-1); Toll-like receptor, TLR; Toll-like receptor 3, TLR3; Toll-like receptor 4, TLR4; Traditional Chinese medicine, TCM; Triple-negative breast cancer, TNBC; Tumor necrosis factor alpha, TNF-α; Tyrosine kinase receptor B, TrkB; Ultraviolet spectroscopy, UV; Vascular endothelial growth factor, VEGF; Wnt/β-catenin, Wnt/β-cat; Xanthine oxidase, XOD.
